# Theoretical Studies on the Adsorption and Degradation of Carbon‐Supported Pt‐ and Pt‐Oxide Nanoparticle Electrocatalysts

**DOI:** 10.1002/smll.202505890

**Published:** 2025-08-28

**Authors:** Julia Bord, Marcin Rybicki, Matthias Baldofski, Christoph Jung, Timo Jacob

**Affiliations:** ^1^ Institute of Electrochemistry Ulm University Albert‐Einstein‐Allee 47 89081 Ulm Germany; ^2^ Freudenberg e‐Power Systems GmbH Bayerwaldstrasse 3 81737 München Germany; ^3^ Freudenberg Technology Innovation SE & Co. KG 69469 Hoehnerweg 2‐4, Weinheim Germany; ^4^ Fraunhofer‐Institute for Mechanics of Materials IWM Wöhlerstraße 11 79108 Freiburg Germany; ^5^ Helmholtz Institute Ulm (HIU) Electrochemical Energy Storage Helmholtz‐Straße 11 89081 Ulm Germany; ^6^ Karlsruhe Institute of Technology (KIT) P.O. Box 3640 76021 Karlsruhe Germany

**Keywords:** atomistic modeling, degradation, detachment, fuel cell, graphite vacancy defects, molecular dynamics, platinum nanoparticles

## Abstract

The performance of electrocatalysts in fuel cells is not only determined by their efficiency under reaction conditions but also by their stability and potential degradation processes. To investigate these factors, molecular dynamics simulation are performed in order to study the adsorption and detachment behavior of 2–3 nm‐sized Pt nanoparticles (NPs) on defective graphite supports with up to 13‐atom large vacancies. Larger NPs show stronger adsorption with increasing vacancy size, particularly for surface‐oxidized NPs (Pt/O ratio 1:1), due to additional van der Waals interactions alongside covalent Pt–C bonds at defect sites. Detachment simulations reveal that larger vacancy defects significantly increase the energy barrier for detachment, especially for non‐oxidized 2 nm NPs, as more Pt atoms remain at the defect after detachment. Surface oxidation reduces detachment barriers by over 50%, while larger NPs generally show lower barriers. In aqueous environments, detachment barriers for non‐oxidized NPs decrease, particularly with larger defects. These findings highlight the critical role of defect size and NP surface oxidation in stabilizing Pt‐NPs on carbon supports, reducing material loss and preserving catalytically active material. Non‐oxidized 2 nm NPs on large defects emerge as promising candidates for stable catalytic applications in both vacuum and aqueous environments, making defect and/or vacancy engineering a promising approach.

## Graphical abstract

Molecular Dynamics simulations reveal the adsorption and detachment behavior of 2–3 nm Pt‐NPs on defective carbon‐supports. Larger vacancy defects enhance adsorption and increase detachment barriers, particularly for non‐oxidized NPs. NP surface oxidation significantly lowers barriers, while aqueous environments further reduce them for non‐oxidized NPs on large defects. These findings provide insights into optimizing Pt‐NPs for stable catalytic applications.

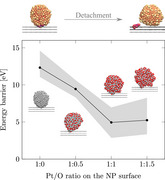

## 1 Introduction

Platinum serves as an exceptionally effective catalyst for a variety of gas‐phase and electro‐catalytic processes. While fundamental research often investigates idealized systems, such as single‐crystal surfaces with well‐defined low‐index orientations, practical applications predominantly rely on supported nanoparticulate catalysts to achieve high activity and durability. This discrepancy underscores the importance of studying realistic catalyst systems and their interactions with the underlying support materials, which often significantly influence their performance and stability.^[^ ^1^, ^2^, ^3^ ^]^

In proton exchange membrane fuel cells (PEMFCs), Pt nanoparticles (NPs) dispersed on carbon‐based supports are the preferred electrocatalyst due to their superior activity and selectivity for the oxygen reduction reaction (ORR).^[^ ^3^, ^4^, ^5^, ^6^ ^]^ However, the long‐term durability of these catalysts is compromised by several degradation mechanisms, including NP agglomeration, detachment from the support material and dissolution.^[^ ^6^, ^7^, ^8^, ^9^, ^10^, ^11^, ^12^, ^13^, ^14^ ^]^ All of these degradation processes represent critical challenges as they directly lead to a loss of active surface area and a subsequent decline in catalytic performance.^[^ ^7^, ^10^, ^13^, ^15^ ^]^

Agglomeration refers to the migration and coalescence of NPs into larger clusters, driven primarily by surface energy minimization and thermally activated diffusion.^[^ ^9^, ^16^ ^]^ This phenomenon, which is often accelerated by high temperatures and weak metal–support interactions, leads to a significant reduction in the number of active sites available for catalysis.^[^ ^9^, ^13^, ^16^ ^]^ Besides this degradation pathway, detachment is another critical issue. This mechanism occurs when NPs are physically separated from their support, often as a result of carbon corrosion, mechanical stress, or weak adhesion between the platinum particles and the support material. The detachment of NPs not only results in the direct loss of active surface area but also contributes to an irreversible decline in catalytic performance, as the detached particles can no longer participate in the electrochemical reactions.^[^ ^6^, ^10^, ^13^ ^]^ Pt dissolution is a key degradation mechanism affecting the stability of fuel cell electrodes, particularly under the operating conditions of PEMFCs. NPs smaller than 2 nm are particularly susceptible due to their high surface energy, which accelerates dissolution through Gibbs–Thomson effects, thereby intensifying stability losses.^[^ ^13^, ^15^ ^]^ At electrochemical potentials within a voltage range of 0.8–1.1V, there is a thermodynamic driving force for surface oxide formation.^[^ ^17^ ^]^ However, at higher potentials (> 1.1V), complete oxidation of the NP surface can occur, leading to the exothermic detachment of small oxidized clusters such as anionic [Pt_6_O_8_]^δ −^ units in aqueous environments.^[^ ^17^ ^]^ These clusters are hydrophilic and stable, contributing to NP fragmentation. Additionally, dissolution is found to be strongly influenced by the coverage of oxygen‐containing adsorbates. Specifically, platinum dissolution is more likely to occur at oxygen coverages below 0.5 ML, as this leads to less subsurface oxygen and the formation of smaller platinum oxide films, which facilitate Pt dissolution.^[^ ^8^ ^]^ Furthermore, the water environment plays a crucial role in these processes, as the interactions between Pt surfaces and water molecules are essential for governing the dissolution kinetics.^[^ ^17^, ^18^ ^]^ The explicit inclusion of water in the simulations allows for more accurate modeling of the dissolution behavior, particularly by accounting for the effects of water dissociation and the subsequent adsorption of species at the Pt‐NP surfaces. Notably, it has been shown that the dissociation of water molecules at Pt‐NP facets, which reconstruct upon oxidation, increases the number of adsorbed species, which in turn slows down the dissolution rates.^[^ ^8^ ^]^ Further studies have shown that elevated operating temperatures significantly accelerate platinum dissolution by increasing the kinetic energy of particles.^[^ ^8^, ^12^ ^]^

The choice and modification of support materials play pivotal roles in mitigating these degradation pathways. Carbon materials offer several advantages as catalyst supports due to their porosity, high specific surface area, and chemical inertness, which facilitate strong metal–support interactions and high catalytic efficiency.^[^ ^19^, ^20^, ^21^ ^]^ Their enhanced electron conductivity makes them particularly suitable for electrochemical applications, including fuel cells, where efficient charge transfer is crucial for the catalytic performance.^[^ ^20^, ^22^ ^]^ Carbon‐based supports – such as carbon black^[^ ^23^ ^]^, carbon nanotubes (CNT)^[^ ^24^, ^25^ ^]^ and graphene^[^ ^26^, ^27^, ^28^ ^]^ or graphite^[^ ^29^, ^30^ ^]^ – are widely used but prone to corrosion, which accelerates Pt‐NP detachment and agglomeration.^[^ ^4^, ^6^, ^31^ ^]^ Advances in support engineering, including defect‐engineered graphite^[^ ^32^ ^]^ or alternative materials such as Fe‐ and N‐codoped carbon,^[^ ^33^ ^]^ have demonstrated potential in enhancing the durability of Pt‐NPs by strengthening metal–support interactions and minimizing oxidation‐induced detachment.

Incorporation of vacancy defects in the support has emerged as an effective approach to enhance the performance and anchoring of Pt‐NPs by significantly modifying their interaction with the support material. Studies indicate that vacancy defects in graphene or carbon nanotubes can serve as strong anchoring sites for metal atoms and clusters, such as Pt^[^ ^34^, ^35^, ^36^ ^]^, Pd^[^ ^37^ ^]^, Ni^[^ ^38^, ^39^ ^]^ and bimetallic Fe_*n*_Pt_*m*_^[^ ^40^ ^]^ systems, due to increased binding energies and enhanced electron transfer at the defect sites. These defects not only immobilize metal atoms effectively, reducing their surface diffusion, but also strengthen the metal–carbon bonds, which mitigates detachment and aggregation under operation conditions.^[^ ^37^, ^41^, ^42^ ^]^

Recent studies have increasingly employed reactive molecular dynamics (ReaxFF‐MD) to investigate Pt–carbon systems at the atomistic level, enabling the modeling of dynamic processes such as cluster–support interaction, oxidation, and structural reorganization under realistic conditions. Sanz‐Navarro et al.^[^ ^43^ ^]^ demonstrated that ReaxFF can effectively capture the adsorption and morphological adaptation of Pt clusters on carbon platelets. Muye et al.^[^ ^44^ ^]^ used ReaxFF‐MD to study methane oxidation on Pt‐decorated functionalized graphene sheet catalysts, showing that support functionalization enhances activity by lowering activation barriers and enabling efficient hydrogen transfer. Chen et al.^[^ ^45^ ^]^ extended this approach by combining ReaxFF‐MD with DFT and in situ kinetics to study CO activation on Pt/CNT systems, highlighting how interfacial charge distributions and oxygen coverage impact catalytic behavior. Xu et al.^[^ ^46^ ^]^ used a multiscale ReaxFF‐MD/DFT approach to investigate Pt NPs on vertically aligned carbon nanofibers (VACNF), finding that strong Pt–C bonding at open carbon edges stabilizes small clusters but also increases the proportion of low‐coordination sites, which weakens ORR activity due to overbinding. Collectively, these studies underscore the potential of ReaxFF‐MD to capture complex structure–reactivity relationships in Pt–carbon systems. However, the role of vacancy‐type defects and the aqueous environment in Pt‐NP detachment and degradation has remained insufficiently addressed. These gaps are the focus of the present study.

Our previous density functional theory (DFT) study has provided valuable insights into the growth behavior of small Pt clusters on graphene supports with vacancy defects. For clusters containing up to 10 Pt atoms, it was shown that larger vacancy defects enhance cluster binding. Additionally, oxidation of the defects significantly impacts cluster stability, with oxidized defects showing weaker Pt binding compared to non‐oxidized systems. Importantly, it was predicted that oxygen adsorption occurs preferentially on the Pt cluster rather than on the support, suggesting that once synthesized, such Pt/C combined materials are less prone to oxidative degradation of the support material.^[^ ^34^ ^]^

To address the above‐described challenges and bridge the gap between idealized models and practical catalyst development, in this work, we have employed reactive molecular dynamics to investigate the adsorption and detachment behavior of Pt‐NPs (2–3 nm) on defective graphite supports. By exploring the interplay between NP size, vacancy defects in the support material, NP surface oxidation, and the role of the aqueous environment, the aim is to provide insights into related degradation processes and potentially help designing more durable Pt‐based catalysts for PEMFCs.

## 2 Results and Discussion

### 2.1 Influence of Defect Size and NP Surface Oxidation

**Figure 1:**
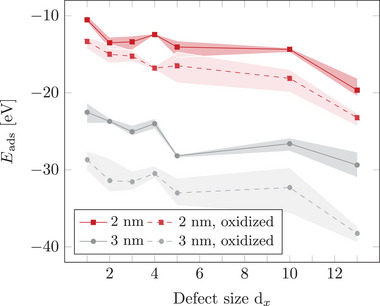
Adsorption energy $E_{\text{ads}}$ (in eV) for 2 and 3 nm large non‐oxidized and oxidized (Pt/O surface ratio 1:1) Pt‐NPs on graphite supports as a function of the vacancy defect size d_*x*_, resulting from the removal of *x* ⩽ 13 carbon atoms.

Vacancies in carbon supports strongly influence metal NP adsorption and impact the overall catalytic performance. In our previous DFT studies we have shown that the support material is critical for the growth of small Pt clusters containing up to 10 Pt atoms.^[^ ^34^ ^]^ Building on these findings, in the present work we investigate larger systems with supported nanoparticulate electrocatalysts over a longer timescale. In particular, we focus on the interaction of graphite with d_*x*_ vacancy defects (*x* ⩽ 13) in the topmost layer (see Figure S1, Supporting Information) and (oxidized) Pt‐NPs with diameters of about 2–3 nm (see Figures S2 and S3, Supporting Information). Stable (oxidized) Pt‐NPs are anchored on each defective graphite model via MD annealing simulations. Details of the annealing procedure are outlined in Section 4. Adsorption energies were evaluated and averaged using Equation (1) for the three most stable NP/defect configurations of each combination. To facilitate the most stable anchoring between the NPs and the defects, the support material is not annealed prior to NP contact, preventing the formation of potential 5‐membered carbon rings and ensuring the availability of dangling C–C σ–bonds for stable Pt– C bonding. **Figure** **1** summarizes the adsorption energies of 2 nm and 3 nm Pt‐NPs as function of the defect size, thereby revealing noteworthy trends.

First, the adsorption process becomes increasingly exothermic with increasing vacancy size in the topmost graphite layer, regardless of the size of the adsorbed non‐oxidized Pt‐NP (solid lines). This trend is consistent with previous findings for small Pt clusters in terms of binding energy.^[^ ^34^ ^]^ As observed for small clusters already, dangling C–C σ‐bonds in the substrate also form strong covalent Pt–C bonds with 2–3 nm NPs, resulting in more stable adsorption. A direct correlation is found between defect size and the number of Pt–C bonds formed (see Figure S4, Supporting Information). The metric increases with growing defect sizes for non‐oxidized 2–3 nm NPs, regardless of NP size. Notably, for the largest defects studied (d_10_ and d_13_), a significant increase in Pt–C bonds compared to smaller defects (up to d_5_) is observed. The slightly lower number of Pt–C bonds in d_13_ compared to d_10_ is explained by the defect geometry: in d_10_, three additional C–C bonds point toward the defect center (Figure S1, Supporting Information), facilitating more Pt–C bond formation. Despite this, the adsorption energy trends in Figure 1 indicate more stable anchoring of Pt‐NPs on d_13_. We assume that this is attributed to steric effects that allow the NP to embed more effectively into d_13_ than into d_10_. Another notable aspect is the less stable adsorption on the d_4_ defect. As previously discussed for small clusters based on the electronic structure, this highly symmetric defect exhibits lower reactivity due to its fully delocalized spin density.^[^ ^34^ ^]^

**Figure 2:**
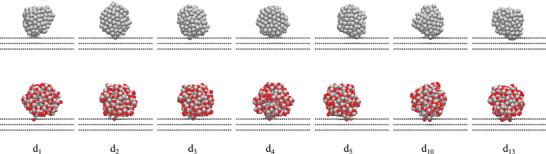
Most stable configurations of anchored 2 nm Pt‐NPs on defective graphite supports d_1_–d_13_. The top row shows non‐oxidized Pt‐NPs anchored on defects with 1–13 missing C atoms in the topmost graphite layer, while the bottom row illustrates oxidized Pt‐NPs (Pt/O surface ratio of 1:1) under the same conditions.

**Figure 3:**
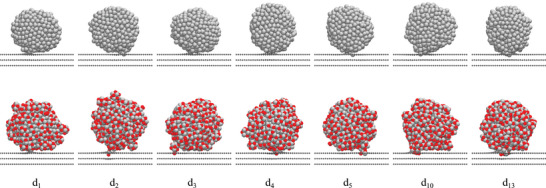
Most stable configurations of anchored 3 nm Pt‐NPs on defective graphite supports d_1_–d_13_. The top row shows non‐oxidized Pt‐NPs anchored on defects with 1–13 missing C atoms in the topmost graphite layer, while the bottom row illustrates oxidized Pt‐NPs (Pt/O surface ratio of 1:1) under the same conditions.

Second, the analysis of Pt–C bonds shows only minor differences between 2 nm and 3 nm NPs, while the adsorption energy values in Figure 1 show a distinct increase in stability for larger NPs. This trend indicates that, beyond Pt–C bond formation, van der Waals (vdW) interactions play a crucial role in stabilizing larger NPs. Due to their larger size and surface area, 3 nm NPs exhibit significantly more vdW interactions with the graphite support, enhancing the interaction between the *d*‐orbitals of Pt atoms and the π‐system of the topmost graphite layer (see Figure S5, Supporting Information). Specifically, the vdW contribution for 3 nm NPs is ≈ 7.5 eV stronger compared to the binding of 2 nm NPs. The observed differences in adsorption behavior between 2 nm and 3 nm NPs are further illustrated in **Figures** **2** and **3**, which show the most stable adsorption structures for each NP and defect size. Certainly, the adsorption structures depend on multiple factors, including the defect size, the initial NP configuration, selected adsorption sites, and simulation conditions. However, generally smaller Pt‐NPs (2 nm) tend to anchor more vertically, especially to larger defects (⩾ d_5_), while 3 nm NPs often align parallel to the graphite substrate, particularly for smaller defects (⩽ d_4_). This parallel alignment maximizes vdW interactions and enhances adsorption stability. However, for larger defects, the size of the vacancy forces 3 nm NPs into a more vertical orientation, leading to a reduction in vdW contributions by ≈ 0.6 eV compared to smaller defects. Despite this reduction, vdW interactions remain a significant stabilizing factor, particularly for the largest NPs studied.

Third, the effect of surface oxidation on Pt‐NPs was investigated (dashed lines in Figure 1). For both 2 and 3 nm NPs, a Pt/O surface ratio of 1:1 was chosen, as this is close to the preferred surface composition and structure at operation potentials of 0.8–1.1 V. The results indicate that oxidizing Pt‐NPs leads to more negative adsorption energy values compared to non‐oxidized NPs of the same size, suggesting stronger anchoring. Averaged over all investigated defect types, surface oxidation enhances the adsorption strength by ≈ 2.9 eV for 2 nm NPs and 6.6 eV for 3 nm NPs. As shown in Figure S5 (Supporting Information), oxidized 2 nm NPs exhibit vdW interactions ≈ 5.8 eV stronger than their non‐oxidized counterparts. The surface oxidation increases the flexibility of the NPs, enabling them to align parallel to the support surface and thereby maximizing vdW interactions, as demonstrated in Figure 2. This trend becomes even more pronounced with 3 nm NPs. In Figure 3, oxidized 3 nm NPs anchor within the defect using a small oxidized Pt cluster, while the remaining NP surface aligns parallel to the support, further enhancing vdW interactions. The larger surface area of 3 nm NPs amplifies the effect of oxidation, resulting in vdW interactions ≈ 13.3 eV stronger compared to non‐oxidized 3 nm NPs. This difference is consistent with the observed adsorption strength, which shows that surface oxidation has a greater stabilizing effect for 3 nm NPs compared to 2 nm NPs. In addition to the reduced number of Pt–C bonds (see Figure S4, Supporting Information), surface oxidation of Pt‐NPs also affects the nature of the bonding with the defective graphite support. Our previous DFT study^[^ ^34^ ^]^ has shown that the binding energy of Pt clusters on defective graphene becomes less exothermic with increasing oxidation of the underlying defects. This trend indicates weaker interaction and supports the conclusion that direct Pt–C bonds are energetically more favorable than the Pt–O–C interactions.

Furthermore, the trend that the adsorption energy values decrease with increasing defect size also applies to oxidized Pt‐NPs.

### 2.2 Degradation through Detachment of NPs from the Underlying Defective Support

One key to enhancing the catalytic activity of platinum NP electrocatalysts and ensuring their long‐term stability lies in elucidating the underlying mechanisms responsible for their degradation. Thus, in the following detachment processes are studied in detail in order to evaluate the influence of defective supports, the degree of surface oxidation of Pt‐NPs, and the impact of an aqueous environment.

A 2–3 nm (oxidized) NP stably‐anchored to a defective graphite support remains immobile during MD simulations at 300 K, even with small defects such as a single missing C atom. The strong anchoring prevents NP agglomeration, enhancing the long‐term stability of Pt catalysts. To this end, the detachment of realistic systems both in vacuum and under aqueous conditions is simulated.

**Figure 4:**
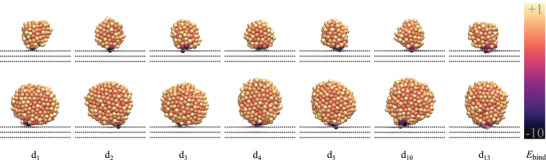
Illustration of the binding energy for individual Pt atoms for the most stable non‐oxidized 2 nm (top row) and 3 nm (bottom row) Pt‐NPs on d_1_–d_13_ defective graphite supports, with 1–13 missing C atoms in the topmost graphite layer. The binding energy is referenced to bulk Pt. Positive values indicate that the Pt atom is less stably bound compared to bulk Pt. Calculation details can be found in Section 4, Supporting Information. Scale is given in eV.

For the detachment simulations, the stably‐adsorbed NPs described in Section 2.1 serve as starting configurations. After an equilibration phase, an additional restraint energy is applied to the center of mass of the corresponding particle in order to force detachment from the support. Further details are provided in the Computational Section. Interestingly, all simulations reveal that after detachment, a small cluster of the NP remains within the defect. Therefore, the strong anchoring between the non‐oxidized Pt‐NPs and the defective graphite support is further investigated by analyzing the binding energy values of each individual Pt atom, see **Figure** **4**. Pt atoms anchored directly in the defect are more strongly bound due to the formation of stable Pt– C σ‐bonds between the NP and the defect. Because of the strong anchoring of the NP with the dangling C–C σ‐bonds, simulations exclusively show the breaking of Pt–Pt bonds within the NP during detachment. The number of remaining Pt atoms in the defect depends on the size of the defect and is analyzed in detail in Section 2.2.2.

Since all NPs in this study, especially the oxidized ones, have a disordered surface, they are not uniformly anchored in the defect, which leads to various detachment processes. For non‐oxidized NPs, the variations in binding energy around the defect (see Figure 4) indicate different levels of barriers depending on the direction of detachment. An exemplary energy diagram depicting the detachment of the most stable oxidized 3 nm NP from the d_13_ defect is presented in Figure S6 (Supporting Information).

To obtain a comprehensive picture of possible detachment processes, detachment simulations were conducted in four distinct directions per NP‐defect configuration. In each case, the NP was detached at a 30° angle relative to the graphite plane, representing out‐of‐plane detachment. The four directions differ in their lateral orientation within the surface plane. For each defect size, the three most stable NP‐defect configurations were selected based on their adsorption energies. From each of these configurations, four detachment simulations (one per direction) were performed, resulting in a total of 12 simulations per system.

The results confirm that no particular direction consistently exhibits the lowest energy barrier, as this depends on factors such as anchoring sites, Pt‐C bond strength (see Figure 4) and the local surface composition near the anchoring point. The direction with the lowest detachment barrier indicates the most likely detachment event.

To avoid considering improbably high barriers and to prevent relying on a single minimum, we selected the lowest detachment barrier for each of the three NP–defect configurations. These values were then averaged to obtain a representative detachment barrier for the system.

A detailed analysis of the detachment angles for the most stable oxidized 3 nm NP on d_13_ shows that the vertical detachment (90°) has significantly higher energy barriers (see Figure S7, Supporting Information). This is due to the deformation of the top graphite layer and the simultaneous breaking of several Pt–Pt bonds, which makes it less likely in realistic systems. In contrast, energy barriers are more than 1.5 eV lower at detachment angles between 30° and 60°. The sequential breaking of bonds at these angles facilitates detachment. Therefore, in our forthcoming studies we concentrate on a 30° angle in order to describe the most likely detachment processes.

However, the determination of energy barriers is challenging due to the complex nature of the detachment process. Each system analyzed in this study exhibits unique characteristics, influenced by NP size, oxygen content, and the specific defect in the graphite support. The initial anchoring configuration varies depending on the local oxidation near the defect, leading to different detachment mechanisms. Additionally, factors such as the detachment angle and direction further influence the analysis, as the NP is not uniformly embedded in the defect. As a result, energy profiles differ in each case. Nevertheless, clear trends emerge across the systems studied, indicating that tendencies – in addition to absolute energy barriers – are decisive for understanding and comparing detachment behavior.

#### 2.2.1 Impact of Surface Oxidation on NP Detachment

We have previously shown that oxidation of defects in the underlying graphene substrate reduces the binding energy between small Pt clusters with up to 10 Pt atoms and the support.^[^ ^34^ ^]^ Extending this concept, we investigate how the surface oxidation of 3 nm Pt‐NPs influences the detachment energy barrier from a graphite support with 13 missing C atoms. The study considers non‐oxidized Pt‐NPs alongside oxidized NPs with Pt/O surface ratios of 1:0.5, 1:1, and 1:1.5, mimicking different surface oxide thicknesses. At higher Pt/O ratios, the NPs begin to dissolve, with small Pt‐oxide clusters detaching from the NP surface. Due to solvent stabilization the detachment of these Pt_6_O_8_ units is slightly exothermic in an aqueous environment.^[^ ^17^ ^]^

**Figure 5:**
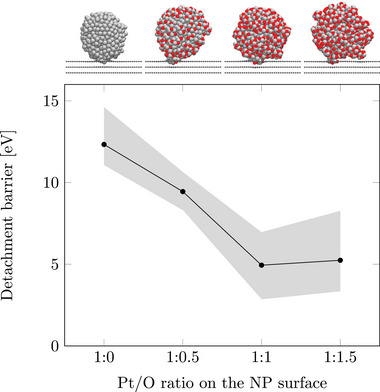
Detachment barriers of a 3 nm Pt‐NP as a function of the Pt/O surface ratio on a d_13_ defect in graphite. The black markers represent the averaged lowest detachment energy barriers from three initial stable NP configurations on the defect. The gray shaded area indicates the range (minimum to maximum) of these lowest barriers. Illustrations of the most stable NP configuration for each oxidation state are shown at the top.

**Figure** **5** shows a systematic reduction in the energy barrier for detachment with increasing degree of surface oxidation. Non‐oxidized NPs exhibit the highest detachment barrier, exceeding 12 eV. Even a slight oxidation (Pt/O surface ratio of 1:0.5) reduces the barrier by around 3 eV. At a Pt/O ratio of 1:1, the detachment barrier plateaus at ≈5 eV, with no significant further reduction observed at higher oxidation levels. This represents a decrease of more than 7 eV compared to non‐oxidized NPs. This behavior can likely be explained by the increasing formation of small oxidized Pt cluster units at the defect site with higher surface oxidation. This trend is exemplarily visualized in Figure 3, showing the most stable structures of non‐oxidized and surface‐oxidized (Pt/O ratio 1:1) Pt‐NPs.

The results show that oxidized NPs are more susceptible to detachment, while non‐oxidized NPs have significantly higher detachment energy barriers. After detachment, all simulations show that a small Pt cluster remains in the defect, emphasizing the strong Pt–C σ‐bonds. In summary, surface oxide formation on the Pt‐NPs critically influences the detachment, and thus the degradation behavior. While a lower degree of surface oxidation is preferred in order to stabilize the Pt‐NPs and to prevent agglomeration, the observed catalytic properties for the ORR might be related to exactly such surface oxides.^[^ ^17^, ^47^ ^]^ Therefore, it might exactly be this interplay of surface oxide formation and reduced stability that fosters the observed catalytic efficiency. However, the presented detachment mechanism of oxidized NPs show reduced barriers, so it can be assumed that they are more likely to detach, which could explain degradation processes usually observed in realistic systems.

#### 2.2.2 Detachment of Pt‐NPs without Solvent

As demonstrated in Section 2.2.1, increasing surface oxidation of the NPs reduces detachment energy barriers. However, while the focus was on the degree of surface oxidation with constant NP and defect sizes, here we systematically analyze the impact of varying defect and NP sizes.

**Figure 6:**
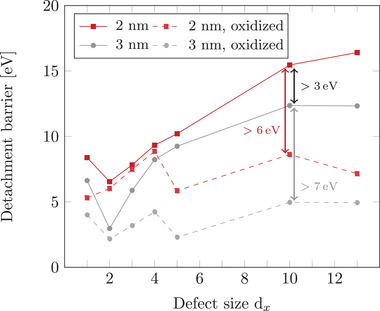
Detachment barriers as a function of the defect size d_*x*_ for 2 nm and 3 nm non‐oxidized and oxidized (Pt/O surface ratio of 1:1) Pt‐NPs. The markers indicate the averaged lowest energy barriers of the three initial most stable NP structures on the given defect. The uncertainties in the detachment barriers are visualized in Figure S9 (Supporting Information).

The solid lines in **Figure** **6** illustrate that larger defect sizes generally lead to higher detachment energy barriers for non‐oxidized 2 and 3 nm Pt‐NPs, with the exception of the single vacancy defect. For this defect, both NP sizes exhibit slightly higher barriers than for the double vacancy defect. For defects with three missing C atoms in the topmost graphite layer, the detachment barrier reaches a similar level as for the single vacancy defect and increases with defect size. Specifically, for 2 nm NPs, the largest defects (d_10_ and d_13_) result in barriers that are over 5 eV higher than for d_5_, indicating a strong interaction between the NP and the defect site. This can be attributed to a good structural match, which in this context refers to the geometric complementarity between the NP and the largest studied vacancy defects in the underlying support. Larger defects allow the NP to embed more deeply into the graphite surface, increasing the contact area and enabling the formation of a greater number of Pt–C σ–bonds with undercoordinated C atoms at the defects. This leads to stronger adsorption and, consequently, higher detachment barriers. This trend of increasing detachment barriers with defect size is further supported by the analysis shown in Figure S8 (Supporting Information), which quantifies the number of Pt atoms remaining embedded in the defect after detachment. The number of residual Pt atoms increases with defect size, which can be attributed to the more stable anchoring of the NP in larger vacancies. These allow for an increased number of Pt–C σ–bonds to form at the NP–support interface (see Figure S4, Supporting Information), thereby strengthening the interaction. A clear correlation can be observed for the studied non‐oxidized 2 nm and 3 nm Pt‐NPs: larger residual Pt clusters correspond to higher detachment barriers. Notably, despite stronger adsorption, 3 nm NPs exhibit lower detachment barriers than 2 nm NPs. This counterintuitive behavior can be attributed to the smaller residual clusters left behind by 3 nm NPs in defects with up to 5 missing C atoms. For the largest defect d_13_, the significantly smaller size of the residual clusters left by 3 nm NPs correlates directly with the observed lower detachment barriers.

The dashed lines in Figure 6 show that surface oxidation (Pt/O surface ratio of 1:1) reduces detachment energy barriers compared to non‐oxidized systems. While this trend was previously shown only for the 3 nm NP on the d_13_ defect, the systematic analysis confirms that it applies to all studied systems. The oxidation causes a shift to lower barriers and a slight alteration in the curve profile. Up to defect size d_4_, oxidized 2 and 3 nm NPs exhibit only slightly lower barriers than non‐oxidized NPs. However, for defects with 5 to 13 missing C atoms, the barrier reduction becomes significant. For example, oxidized 2 nm NPs exhibit a barrier reduction of over 50 % for the largest defect, while oxidized 3 nm NPs show a reduction of ≈ 7 eV for the three largest defects. This behavior can be attributed to the initial structures of the oxidized NPs, which exhibit fewer Pt–C bonds compared to non‐oxidized Pt‐NPs (see Figure S4, Supporting Information).

In summary, larger defect sizes lead to more pronounced differences in detachment barriers between non‐oxidized and oxidized systems. Defects with at least 10 or 13 missing C atoms serve as suitable substrates for 2–3 nm NPs, preventing degradation due to their strong interaction. Using non‐oxidized NPs further hinders degradation, maintaining the catalytic activity over extended periods.

#### 2.2.3 Influence of an Aqueous Environment on the Detachment of Pt‐NPs

**Figure 7:**
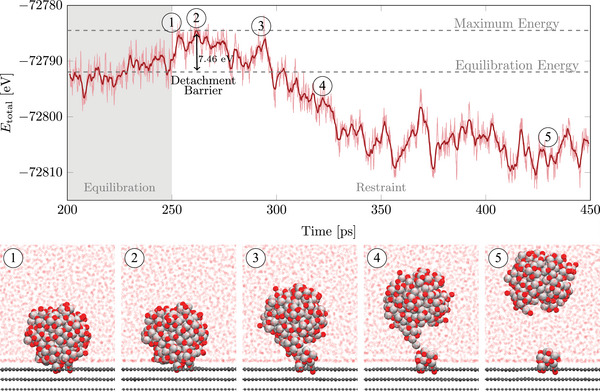
Exemplary energy profile showing the last 50 ps of the equilibration phase and the detachment process of a 2 nm oxidized (Pt/O ratio of 1:1 on the NP surface) Pt‐NP from the d_13_ defective graphite support in aqueous environment. The raw energy data (light red) and the smoothed energy curve (dark red) are both shown. Snapshots illustrate key stages during the detachment simulation.

In Section 2.2.2, we demonstrated that oxidized Pt‐NPs significantly lower the detachment barriers for 2–3 nm NPs. Building on this, we now investigate the effect of an aqueous environment on these detachment simulations. Due to the high computational cost, we have focused specifically on 2 nm Pt‐NPs. The same initial structures from the previous Section were solvated and equilibrated before running detachment simulations. During the detachment process, both the NP and the residual part left in the defect are additionally solvated. An exemplary energy diagram for a 2 nm oxidized NP on a d_13_ defective graphite support in an aqueous environment is shown in **Figure** **7**. The first snapshot depicts the system at the end of the equilibration phase. Subsequently, the restraining condition is applied, with the second snapshot illustrating the moment when the maximum total energy is reached. As the detachment process progresses, the structure shown in the third snapshot is observed, leading to the detachment of the NP while leaving behind a residual part of the NP within the defect, as depicted in snapshot 4. Finally, snapshot 5 presents the fully detached NP in the aqueous environment.

**Figure 8:**
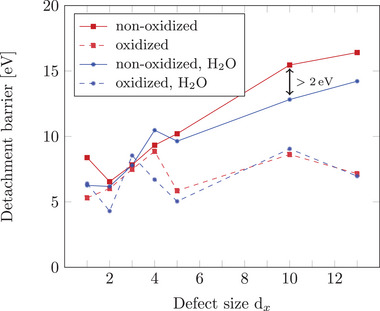
Comparison of detachment barriers in vacuum and aqueous environment as a function of the defect size d_*x*_ for 2 nm non‐oxidized and oxidized (Pt/O ratio of 1:1 on the NP surface) Pt‐NPs. The markers indicate the averaged minimum energy barriers of the 3 initial most stable NP structures on the given defect. The uncertainties in the detachment barriers are visualized in Figure S10 (Supporting Information).

Following the solid lines in **Figure** **8**, only a slight difference in detachment barriers is observed between vacuum and aqueous conditions for non‐oxidized Pt‐NPs with defects involving up to 5 missing C atoms. For these small defects, we also observe minor fluctuations in the detachment barriers rather than a strictly monotonic trend. This behavior likely arises from the limited number of anchoring sites and the reduced geometrical flexibility of small vacancies, which makes the system highly sensitive to the initial NP configuration. Even subtle variations in atomic arrangements can influence the stability of the residual Pt cluster after detachment and, thus, affect the calculated barrier. For larger defects (d_10_ and d_13_), however, a significant reduction in the detachment barrier is observed in an aqueous environment. This behavior arises from the fact that smaller defects anchor a relatively small cluster within the defect, meaning only a limited NP surface area becomes additionally solvated during detachment. In contrast, larger defects expose a greater portion of the NP's surface at the detachment point to the surrounding water, leading to a stronger stabilization effect. As a result, the detachment barrier is lowered by over 2 eV for the largest defects studied.

In contrast, oxidized NPs do not show this pronounced influence of the aqueous environment. For these systems, the detachment barriers in water, particularly for defects with 5 or more missing C atoms, are nearly identical to those observed in vacuum. Since the detachment transition state involves minimal polarity or charge separation, little difference between vacuum and water is expected, as water mainly stabilizes polar or charged states. In summary, the aqueous environment plays a more critical role in the detachment process of non‐oxidized Pt‐NPs than it does for oxidized NPs. The observed trend can be rationalized by considering the relative interaction strengths between water molecules and Pt or graphite. Previous DFT studies report the static interaction energy of a single water molecule with Pt(111) to be in the range of −0.40 ^[^ ^48^ ^]^ to −0.464 eV^[^ ^49^ ^]^, whereas the water – graphite interaction is significantly weaker, with an interaction energy of ≈ −0.13 eV^[^ ^50^ ^]^. From a simplified perspective – neglecting dynamic and many‐body effects – this difference implies that the detachment of a Pt–NP is accompanied by additional stabilizing Pt–water interactions, effectively lowering the energy barrier. In contrast, the interaction of water with oxidized Pt is markedly weaker – similar to its interaction with graphite. This trend is confirmed by our MD simulations: while liquid water reduces the detachment barrier for Pt, it has no significant effect on oxidized Pt. The dynamic hydration effects of Pt and PtO NPs were previously investigated by our group^[^ ^17^ ^]^. These effects are inherently complex and strongly depend on particle shape, which varies with oxygen content. At low oxygen levels, the solvation energy increases with oxygen content, reaching a maximum at an O/Pt ratio of ≈0.2. Beyond this point, the solvation energy becomes more stabilizing, which could also be influenced by the increasing number of atoms in the system. These results highlight the complex interplay of energetic and entropic contributions in the ternary Pt/C/water system, the detailed exploration of which is beyond the scope of this work.

## 3 Conclusion 

Based on reactive force field simulations in the present study, we provide comprehensive insights into the adsorption and detachment behavior of 2–3 nm Pt‐NPs on defective graphite support models with up to 13 missing C atoms, focusing on the effects of Pt‐NP size, degree of oxidation and support material defects.

The findings confirm that the adsorption energy of 2–3 nm Pt‐NPs decreases with increasing vacancy defect sizes in the support, consistent with previous DFT studies on smaller Pt clusters with up to 10 Pt atoms.^[^ ^34^ ^]^ The adsorption energy is influenced by both the size and oxidation degree of the Pt‐NPs. Larger NPs exhibit lower adsorption energy values due to vdW contributions, originating from the interactions between the *d*‐orbitals of Pt atoms and the π‐system of the graphite support. Furthermore, surface‐oxidized NPs (Pt/O surface ratio 1:1) show significantly more exothermic adsorption compared to non‐oxidized NPs of the same size.

In terms of degradation, our simulations revealed that vacancy defects are critical for stabilizing Pt‐NPs by preventing their mobility on the carbon surface. Even small defects effectively trap NPs by creating strong C–Pt σ‐bonds, mitigating agglomeration and enhancing long‐term stability. Detachment of the NPs from the surface defect results in its rupturing, leaving a small part of the NP inside the vacancy. Larger defects (10 and 13 missing C atoms) exhibit particularly high energy barriers for detachment, especially for 2 nm NPs. This is likely due to a favorable size match between the NP diameter and the dimensions of the defects. Oxidized Pt‐NPs exhibit lower detachment barriers compared to non‐oxidized NPs, suggesting that a higher Pt/O surface ratio facilitates easier detachment. While larger vacancy defects correspond to higher detachment barriers for 2–3 nm Pt‐NPs, larger NPs generally lead to lower detachment barriers. This behavior correlates with the number of Pt atoms remaining in the defect after detachment. Specifically, larger defects tend to retain larger Pt clusters, whereas 3 nm NPs leave slightly smaller clusters behind compared to 2 nm NPs on the same support.

Surface oxidation of NPs significantly reduces detachment barriers, with oxidized NPs showing more than 50 % lower barriers for the largest defects d_10_ and d_13_, compared to non‐oxidized NPs of the same size. Additionally, in aqueous environments, the detachment barriers for non‐oxidized 2 nm NPs slightly decrease for defects with up to 5 missing C atoms. For the largest studied defects, however, a significant reduction in detachment barriers of more than 2 eV is observed due to solvation effects stabilizing the detachment point. In contrast, the aqueous environment has only a minimal effect on the detachment barriers of the oxidized NPs, which underlines the stronger role of water in the detachment process of the non‐oxidized Pt‐NPs.

In summary, these results underscore the crucial role of defect size and NP surface oxidation in the adsorption behavior and degradation resistance of Pt‐NPs on graphitic supports. Large vacancy defects in the graphite substrate, combined with minimally oxidized Pt‐NPs, provide the most stable configuration, reducing degradation through detachment and enhancing catalytic longevity. These findings highlight the potential of systems involving non‐oxidized 2 nm Pt‐NPs on large vacancy defects (d_10_ and d_13_) in both vacuum and aqueous environments as promising candidates for experimental efforts. Such systems could prevent severe material loss and preserve catalytically active surfaces, thereby guiding the development of more durable and efficient catalytic materials. To achieve this experimentally, strong oxidative synthesis or operating conditions should be avoided, as they may promote Pt surface oxidation and introduce oxidized defects, both of which lower NP stability. In particular, wet‐chemical synthesis methods involving extensive oxidation, such as Hummer's method^[^ ^51^ ^]^, should therefore be reconsidered when long‐term catalyst performance is a priority.

## 4 Computational Section

All MD calculations were performed using the ReaxFF reactive force field method in the Amsterdam Density Functional Suite Version 2021.102.^[^ ^52^ ^]^ ReaxFF is a force field dependent on bond orders, that excels in accurately representing the breaking and forming of bonds. The potential energy depends on the bond order, which is dynamically updated during each MD simulation step based on interatomic distances. In addition to binding interaction terms, ReaxFF describes non‐bonding terms with Coulomb and vdW potentials.^[^ ^53^, ^54^ ^]^ This enables the simulation of chemically reactive systems, including processes such as water dissociation, hydroxyl formation, and surface oxidation. The Pt/O/H/C force field was used for all simulations.^[^ ^55^ ^]^ Details regarding the validation of the employed reactive force field are provided in Section S10 (Supporting Information). Cuboctahedral Pt‐NPs with diameters of ≈2 and 3 nm were generated by cutting from an *fcc*‐Pt crystal with a lattice constant of 3.95 Å^[^ ^17^, ^56^ ^]^ using the nanocut python tool.^[^ ^57^ ^]^ This NP size range was chosen due to the NPs' relevance for catalytic applications, particularly in fuel cells.^[^ ^13^, ^58^ ^]^ Thermodynamically stable oxidized Pt‐NPs were obtained via ReaxFF‐based grand canonical Monte Carlo (GCMC) calculations. The GCMC algorithm, as developed by Senftle et al.,^[^ ^59^, ^60^ ^]^ randomly inserts, deletes, or moves oxygen atoms within the simulation box while keeping the number of Pt atoms constant. Detailed compositions of the (oxidized) Pt‐NPs are provided in the Table S1 (Supporting Information). In all cases, the oxidation of the Pt‐NPs was carried out prior to their contact with the graphite supports.

A timestep of 0.25 fs was employed during all MD simulations. The Velocity–Verlet algorithm and a Berendsen thermostat (*NVT* ensemble) with a temperature damping constant of 100 fs controlled the system temperature. Charge equilibration was performed using the electronegativity equilibration method (EEM). An elastic wall was applied to limit particle motion in the *z*‐direction, especially for simulations in an aqueous environment. The simulation boxes varied depending on NP size. For 2 nm NPs, the box dimensions were approximately 52 × 51 × 70 Å^3^, while for 3 nm NPs, the box size was 158 × 137 × 100 Å^3^.

Annealing simulations were first performed to obtain stable non‐oxidized and oxidized NPs (see Table S1 and Section S11, Supporting Information for details). A second annealing step was then carried out to anchor the respective non‐oxidized or oxidized NPs in different orientations on defective graphite supports, which consists of three layers. Graphite was chosen as the support material due to its structural stability and layered nature, which allows for a realistic representation of catalyst–support interactions, including subsurface carbon effects that significantly influence the detachment process. In contrast, single‐layer graphene or reduced graphene oxide models tend to deform during detachment simulations, artificially affecting energy barriers. Initially, the defective carbon support was energy‐minimized, with C atoms in the bottom layer and atoms at the edges of the periodic cell fixed. During annealing, the NP/defect systems underwent three treatment phases: During the initial annealing phase, the system underwent a controlled heating process, ramping up from 300 to 600 K over 25 ps. In the subsequent equilibration phase, the system temperature was sustained at 600 K over 50 ps. The third phase involved a gradual cooling of the systems to 0 K over 100 ps, followed by an energy minimization phase lasting 5 ps. Ten different anchoring points on the NP surface were selected, and the three most stable configurations for each NP and defect combination were used for further simulations. For a Pt‐NP adsorbed on defective graphite, the adsorption energy $E_{\text{ads}}$ is referenced to the energy of the corresponding annealed graphite support $E_{\text{graphite}}$ and the annealed isolated Pt‐NP with total energy $E_{\text{NP}}$:

(1) $$E_{\mathrm{ads}}=E_{\mathrm{NP}/\mathrm{graphite}}-E_{\mathrm{graphite}}-E_{\mathrm{NP}}$$

where $E_{\text{NP/graphite}}$ represents the total energy of the adsorbed Pt‐NP/graphite system. Adsorption energy values were averaged across the three most stable structures of each combined NP/defect system. Thus, the more negative *E*_ads_ is, the stronger is the binding.

Detachment simulations were performed at 300 K using constrained MD to investigate energy changes during NP detachment for the three most stable adsoption structures of each NP/defect system. The systems were pre‐equilibrated in vacuum for 200 ps (800000 iterations) and in an aqueous environment (density ρ_H__2__O_ = 1.0 g cm^−3^) for 1000 000 iterations. A restraining condition was then applied, forcing the NP to detach from the defect over at least 400 000 iterations. The pulling velocity was set to 0.0001 Å fs^−1^. The constraint was applied to three central atoms of the Pt‐NP, with bond distances between the central atom and its 12 nearest neighbors fixed to distribute the restraint energy. The bond restraint is implemented in the ReaxFF potential by introducing an additional restraint energy, *E*_restraint_, between two atoms *i* and *j*. This energy term helps maintaining the interatomic distance at a defined value, *R*_restraint_, with its strength controlled by two parameters, *f*_1_ and *f*_2_, as described in Equation (2).^[^ ^61^ ^]^ During each iteration step along the MD simulation, the actual interatomic distance, *R*_*ij*_, is updated, thereby applying the restraint energy to displace the Pt‐NPs from the underlying defects, allowing the system to overcome the detachment barrier.

(2) $$E_{\mathrm{restraint}}=f_{1}\cdot \left[1.0-e^{-f_{2}\cdot {\left(R_{ij}-R_{\mathrm{restraint}}\right)}^{2}}\right]$$

For the constrained simulations carried out in this work, a constant *f*_1_ value of 500 kcal mol^−1^ was used, while *f*_2_ was set to 0.25 Å^−2^. Energy barriers were determined by comparing the average total energy during the final 200 000 iterations of pre‐equilibration with the total energy at the point of detachment. Detachment was consistently found to be a kinetically activated process across all systems. Further details regarding the computational cost are provided in Section S12 (Supporting Information).

## Conflict of Interest

The authors declare no conflict of interest.

## Supporting information

Supporting Information

## Data Availability

The data that support the findings of this study are available from the corresponding author upon reasonable request.
