# Supplementary material for: Theoretical Studies on the Adsorption and Degradation of Carbon‐Supported Pt‐ and Pt‐Oxide Nanoparticle Electrocatalysts
Source: Small. 2025 Aug 28;21(47):e05890. doi: 10.1002/smll.202505890 (PMC12658937; doi:10.1002/smll.202505890)
Supplement: Supplementary file 1 — Supporting Information [file SMLL-21-e05890-s001.pdf]

NANO · MICRO
small

Supporting Information

for *Small*, DOI 10.1002/smll.202505890

Theoretical Studies on the Adsorption and Degradation of Carbon-Supported Pt- and Pt-Oxide Nanoparticle Electrocatalysts

Julia Bord, Marcin Rybicki, Matthias Baldofski, Christoph Jung and Timo Jacob**

Supporting Information

Theoretical Studies on the Adsorption and Degradation of Carbon-Supported Pt- and Pt-Oxide Nanoparticle Electrocatalysts

Julia Bord, Marcin Rybicki, Matthias Baldofski, Christoph Jung, Timo Jacob**

S1 Support Models

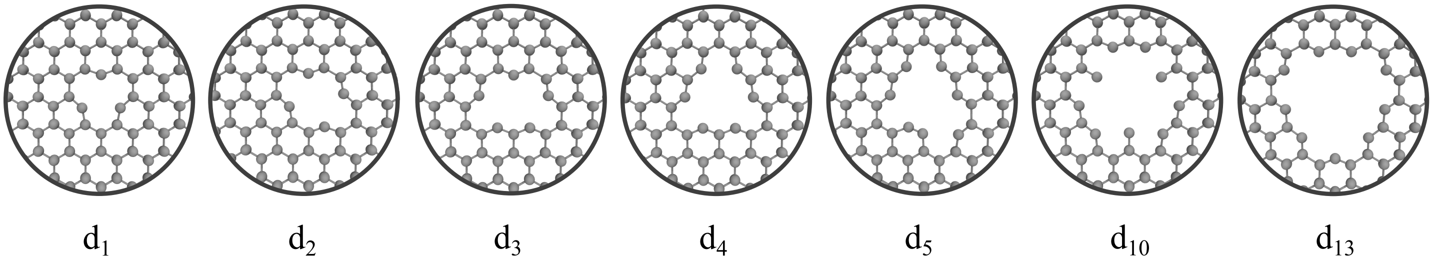

Figure S1: Configurations of the topmost graphite layer with various defect sizes d_x resulting from the removal of $x \leq 13$ C atoms.

S2 NP Models

To generate the most stable non-oxidized and oxidized Pt-NPs, 2 and 3 nm cuboctahedral NPs were oxidized using GCMC simulations. Subsequently, two structures were selected for the 2 nm NP: the non-oxidized Pt structure and the NP with a Pt/O surface ratio of 1:1. For the 3 nm NP, Pt/O ratios of 1:0, 1:0.5, 1:1, and 1:1.5 were chosen for further simulations in this study. Details on the composition of the NPs are provided in Table **S1**. To obtain stable initial structures for the adsorption of these NPs on the defective graphite models, the generated NPs underwent annealing (details can be found in Section S11). The resulting structures are depicted in Figure **S2** and Figure **S3**.

Table S1: Composition and annealing temperatures of all Pt-NPs.

NP size [nm]	Pt atoms No.	Pt surface atoms No.	O atoms No.	Pt/O ratio (surface)	O/Pt ratio (NP) [%]	annealing temperature [K]
2	201	122	0	1:0	0.00	3000
2	201	122	122	1:1	0.61	800
3	711	306	0	1:0	0.00	3000
3	711	306	151	1:0.5	0.21	2000
3	711	306	306	1:1	0.43	800
3	711	306	459	1:1.5	0.65	600

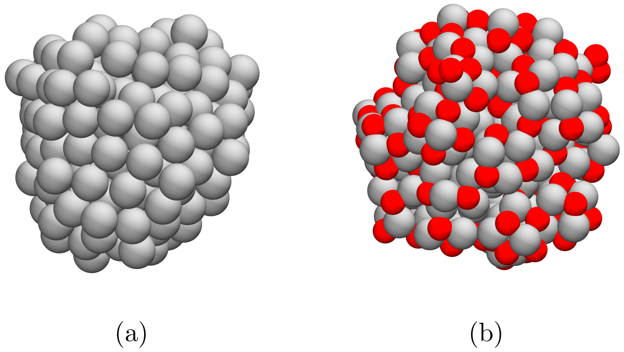

Figure S2: Configurations of stable 2 nm NPs with an Pt/O ratio on the NP surface of (a) 1:0 and (b) 1:1.

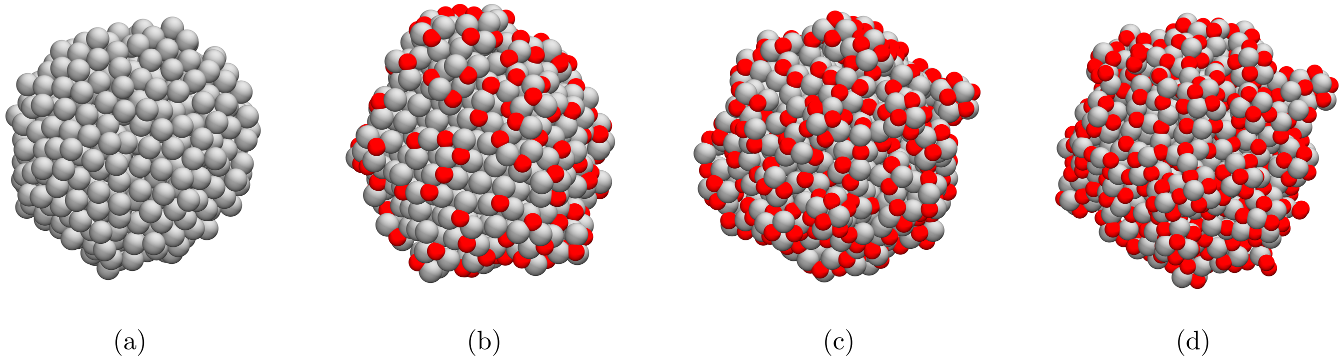

Figure S3: Configurations of stable 3 nm NPs with an Pt/O ratio on the NP surface of (a) 1:0, (b) 1:0.5, (c) 1:1 and (d) 1:1.5.

S3 Initial Adsorbed Structure Analysis

S3.1 Pt–C Bond Analysis

The number of Pt–C bonds in the three most stable adsorbed Pt-NP systems on various defective graphite supports was counted and averaged (see Figure **S4**). A cutoff distance of 2.1 Å was chosen for identifying Pt–C bonds. As Pt–C bonds in clusters show a maximum bond length of 1.99 Å [1], the chosen cutoff is reasonable and ensures the inclusion of all formed Pt–C bonds.

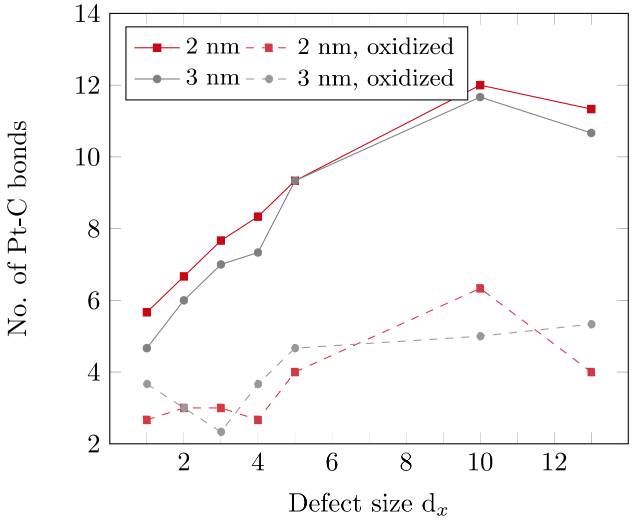

Figure S4: Averaged number of Pt–C bonds for the three most stable structures of each NP/defect combination.

S3.2 Contribution of Pt–C vdW Interactions

The approximate contributions of Pt–C vdW interactions of the three most stable initial adsorption structures on various defective graphite supports were averaged (see Figure **S5**). As described in Section S3.1, all Pt atoms with a Pt–C bond length ≤ 2.1 Å are considered covalently bound to the support. For oxidized structures, a cutoff distance of 1.75 Å was used to identify oxygen atoms covalently bound to the support. The vdW interaction contribution, E_{vdW}, between Pt and C atoms in NPs stably adsorbed on the defective substrates is calculated using Equation S1.

$$E_{\text{vdW}} = E_{\text{NP without covalent bound atoms/graphite}} - E_{\text{graphite}} - E_{\text{NP without covalent bound atoms}} \tag{S1}$$

Here, $E_{\text{NP without covalent bound atoms/graphite}}$ represents the total energy of a single-point calculation of the adsorbed Pt-NP on the graphite support, where all Pt and O atoms covalently bound to the support have been removed to exclude any covalent energy contributions. To isolate the vdW energy contribution, this structure is devided into two subsystems. First, the total energy value of the graphite support E_{graphite} and second, the total energy value of the NP without the covalently bound Pt and O atoms $E_{\text{NP without covalent bound atoms}}$ are subtracted. Both energy values are determined by single-point calculations.

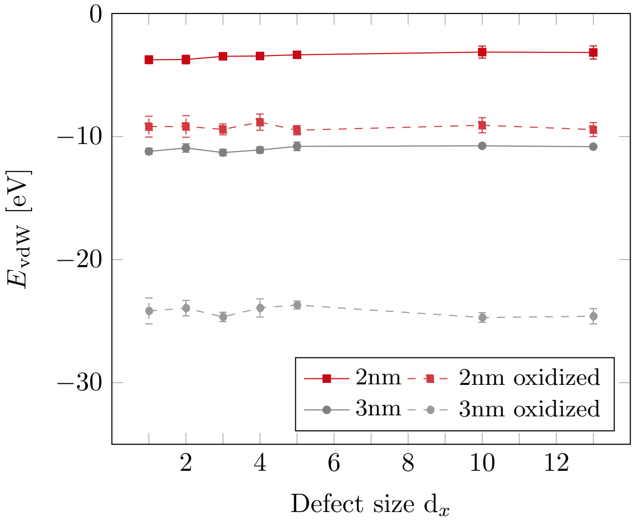

Figure S5: VdW interaction energy values E_{vdW} in eV for 2 and 3 nm large non-oxidized and oxidized (Pt/O surface ratio 1:1) NPs on graphite supports with various vacancy defects d_x, resulting from the removal of $x \leq 13$ C atoms.

S4 Adsorbed Pt-NP Structure Analysis

To calculate the binding energy $E_{\mathrm{b,Pt}_i}$ of each individual Pt_i atom in the supported NP, Equation S2 is used.

$$E_{\mathrm{b,Pt}_i} = E_{\mathrm{NP/graphite}} - E_{\mathrm{NP\ without\ Pt}_i\mathrm{/graphite}} - E_{\mathrm{Pt}} \tag{S2}$$

The binding energy of a specific Pt_i atom is calculated by using the total energy of the supported Pt-NP system and subtracting the energy $E_{\mathrm{NP\ without\ Pt}_i\mathrm{/graphite}}$ of the system with that particular Pt_i atom removed, as well as the energy E_{Pt} of one bulk Pt atom.

S5 Energy Diagramm for the Detachment of an Oxidized 3 nm NP from a d_{13} Defect

Figure **S6** exemplifies the energy profile for the detachment of an oxidized 3 nm NP from the d_{13} defective graphite support. The system is equilibrated for the first 200 000 fs. Then, an additional restraint energy is applied to detach the NP at a 30° angle relative to the graphite surface. During the final 50 000 fs, this additional force is removed, and the system is equilibrated again. Once the additional restraint energy is applied to the NP, the system energy increases as the NP begins to detach, reaching its peak just before complete removal, leaving behind a small cluster within the defect. After detachment, the energy values decrease again. However, each system analyzed in this study exhibits unique characteristics, resulting in different energy profiles for each detachment simulation, depending on the exact detachment mechanism. To evaluate the detachment barrier, the curve is smoothed. The smoothing of the curve is performed using a window of 2000 data points, corresponding to a time span of 500 fs. This choice of window size effectively reduces noise in the data while highlighting the underlying trend. The energy barrier height is calculated as the difference between the energy maximum observed during the NP detachment and the average energy value of the prior equilibration phase.

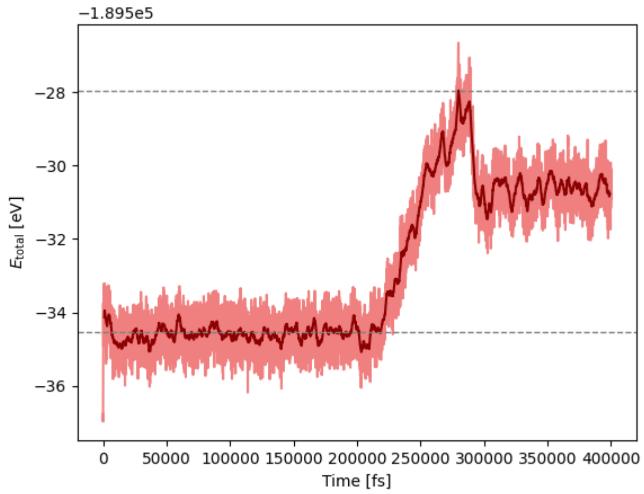

Figure S6: Detachment barrier of the most stable oxidized 3 nm NP (Pt/O ratio of 1:1 on the NP surface) from a d_{13} defective graphite support model. The restraining condition is applied after 200 000 fs.

S6 Energy Barrier of an Oxidized 3 nm NP from a d_{13} Defective Graphite Model as a Function of the Detachment Angle

To understand the effect of the detachment angle, the most stable 3 nm NP on d_{13} graphite is examined, and the detachment barrier is evaluated as a function of the detachment angle between 30° and 90° relative to the defective graphite support plane.

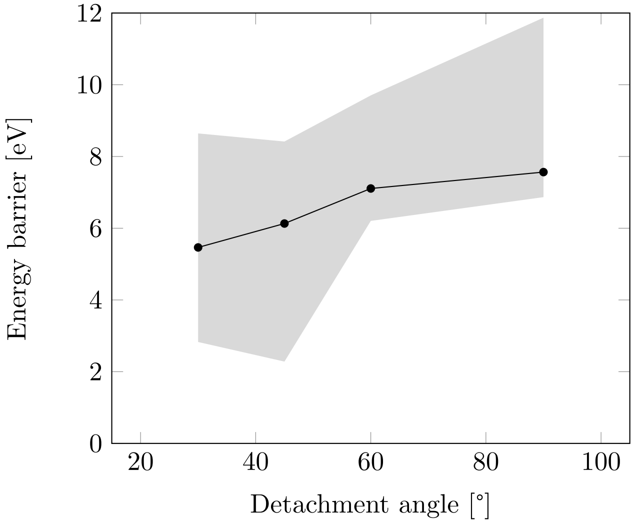

Figure S7: Energy barriers as a function of the detachment angle for an 3 nm large oxidized NP (with a Pt/O ratio of 1:1 on the NP surface) from a d_{13} defective graphite model. The markers indicate the averaged minimum energy barriers of the three initial most stable NP structures. The shaded area additionally represents the range between the minimum and maximum of these lowest barriers.

S7 Remaining Pt Cluster within the Defect after Detachment

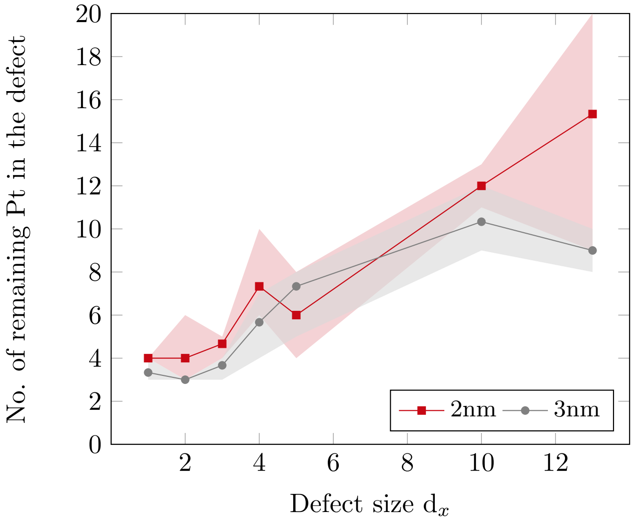

Figure S8: Number of Pt atoms remaining in the defects after detachment of non-oxidized 2 and 3 nm Pt-NPs. The markers represent the average number of Pt atoms remaining in the defect across the three most stable initial NP structures. The shaded area indicates the range between the minimum and maximum number of Pt atoms remaining in the defect for simulations with the lowest energy barriers.

S8 Detachment of 2 and 3 nm (Oxidized) Pt-NPs from d_1-d_{13} Defective Graphene Supports in Vacuum

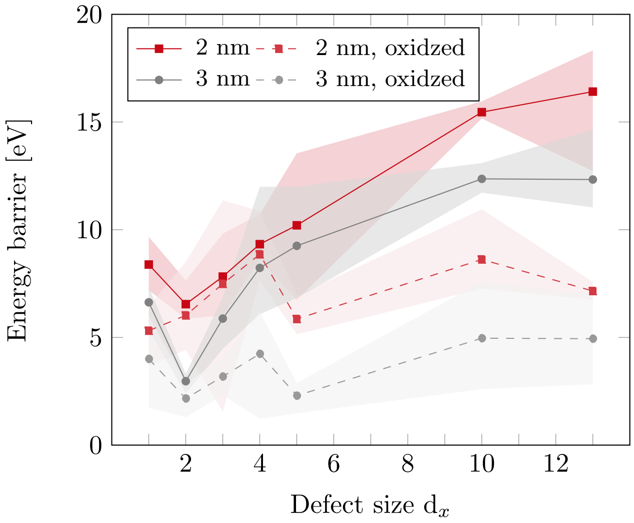

Figure S9: Detachment barriers as a function of the defect size d_x for 2 and 3 nm non-oxidized and oxidized (Pt/O surface ratio of 1:1) Pt-NPs. The markers indicate the averaged lowest energy barriers of the three initial most stable NP structures on the given defect. The shaded area additionally represents the range between the minimum and maximum of these lowest barriers.

S9 Influence of an Aqueous Environment on the Detachment of Pt-NPs

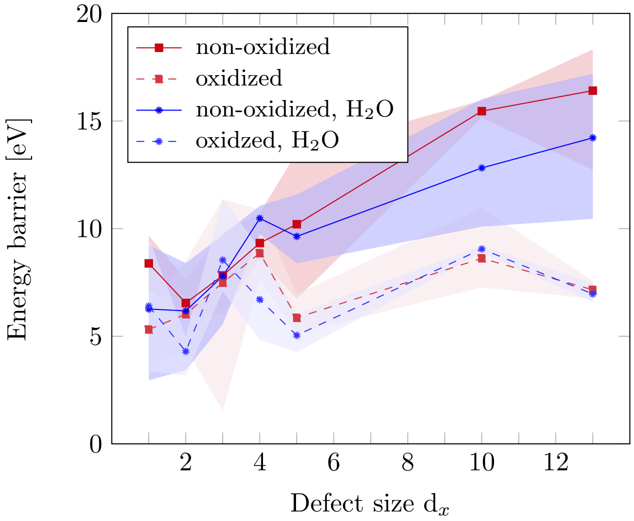

Figure S10: Comparison of detachment barriers in vacuum and aqueous environment as a function of the defect size d_x for 2 nm non-oxidized and oxidized (Pt/O ratio of 1:1 on the NP surface) Pt-NPs. The markers indicate the averaged minimum energy barriers of the three initial most stable NP structures on the given defect. The shaded area additionally represents the range between the minimum and maximum of these lowest barriers.

S10 Validation of the ReaxFF Force Field for Pt/C Systems

To assess the suitability of the employed ReaxFF force field[2] for modeling Pt cluster interactions with defective graphene, we performed a systematic validation by comparing ReaxFF-calculated binding energies with reference data obtained from DFT. The DFT results are taken from our previous work[3], where Pt_n clusters ($n = 1$–10) were grown on graphene substrates containing vacancy defects d_x with $x = 0-5$ missing C atoms. For the validation, we employed the same atomic structures of Pt_n clusters with $n = 4$–10 as used in the DFT calculations, and subjected them to energy minimization using the ReaxFF force field. This range was chosen as it corresponds more closely to the structural motifs relevant for the 2–3 nm NPs investigated in the MD simulations. Energy minimizations using ReaxFF were considered converged when the root mean square gradient (RMSG) of atomic forces dropped below $2.5\,\mathrm{kcal\,mol^{-1}\,Å^{-1}}$, which corresponds to a standard convergence threshold for ReaxFF geometry relaxations in extended systems. The resulting binding energies per Pt atom are plotted in Figure **S11**, where solid lines represent the DFT results and dashed lines indicate the corresponding ReaxFF values. The comparison reveals that the ReaxFF predictions are in good agreement with the DFT data, particularly for the pristine graphene surface (d_0) and defects up to 3 missing C atoms (d_1-d_3). In these cases, ReaxFF reproduces the DFT trend consistently across the entire range of considered cluster sizes. For larger vacancy defects (d_4 and d_5), slightly larger deviations are observed for small-to-intermediate cluster sizes ($n = 4$–5), but the agreement improves for larger clusters ($n \geq 6$). This is expected, as the binding of very small clusters is more sensitive to local coordination environments and electronic effects, which are more accurately captured by quantum mechanical methods. For the largest investigated cluster size (Pt_{10}), the deviation between ReaxFF and DFT binding energies remains below 0.17 eV per Pt atom for defects up to d_4, and reaches approximately 0.23 eV for the most strongly defective case (d_5), which is within the expected accuracy range for reactive force fields. Moreover, the ReaxFF binding energy trends across different defect sizes are consistent with the DFT results, correctly reproducing the relative stability sequence. These findings confirm the reliability of the chosen ReaxFF parameter set for modeling Pt–C interactions under realistic conditions.

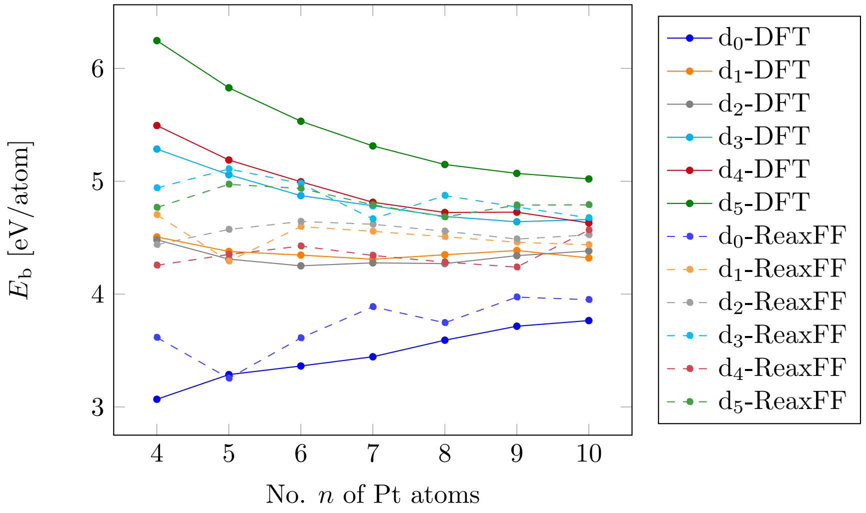

Figure S11: Comparison of binding energie values E_b in eV/Pt atom for Pt_n cluster adsorption on graphene supports with vacancy defects d_x obtained using DFT (solid lines) and ReaxFF (dashed lines). The DFT data are reproduced from Ref.[3] without modification. The ReaxFF results were obtained by energy minimization of the same structures used in the DFT study.

To further support the applicability of the ReaxFF force field under reactive and oxidative conditions, we summarize its prior validation and use in oxidative Pt systems below. The employed ReaxFF force field[2] builds upon the Pt/O parameterization developed by Fantauzzi et al.[4], which was originally trained for oxidation processes on Pt surfaces. These Pt/O parameters were extended to a Pt/O/H force field by introducing new Pt–H interactions while keeping the Pt–Pt and Pt–O terms fixed to ensure full transferability with the original Pt/O force filed. Details on the inclusion of the Pt/H parameters can be found

in Ref.[2]. The underlying Pt/O force field[4] has been successfully applied and validated in prior studies targeting oxidative environments. Notably, Kirchhoff et al.[5] employed this ReaxFF potential within a grand-canonical Monte Carlo framework to simulate the electrochemical oxidation of Pt-NPs (2–4 nm) under realistic operating conditions. Their simulations reproduced the formation of thermodynamically stable surface oxides around 0.8–0.8 V vs. SHE-values consistent with experimental fuel cell conditions - and predicted particle degradation pathways via the formation of $[Pt_6O_8]^{4-}$ clusters at higher potentials. These results were supported by complementary DFT calculations, highlighting the relevance of this force field for studying oxidative Pt degradation. Furthermore, the Pt/O ReaxFF force field has undergone extensive validation for both extended and nanoparticulate Pt systems. As reported by Kirchhoff et al., the force field accurately describes the formation and stability of oxidized surface phases on Pt(111), including surface-buckled and subsurface oxide structures, which were corroborated by in situ XPS measurements[6,7]. Its transferability to NP systems was confirmed by Kirchhoff et al. through a two-step validation: (i) energy trends for model NPs with different morphologies and sizes (1–10 nm) were shown to be in agreement with results from DFT and quantum-corrected Sutton–Chen-type potentials[4,8]; (ii) the formation energies of small Pt clusters (< 100 atoms) reproduced reference DFT data appropriate. Notably, for catalytically relevant particle sizes around 3 nm, the force field predicted energetics and structural trends consistent with experiment and theory. These findings confirm the reliability of the applied Pt/O/H/C ReaxFF force field in describing both Pt/C interactions and oxidation-driven structural transformations in Pt-NPs under reactive and electrochemical conditions, thereby providing a solid foundation for its application in the present study, where Pt-NPs are modeled on carbon supports with various defect structures.

S11 Annealing Process of Pt-NPs

The 2 nm and 3 nm cuboctahedral Pt-NPs were subjected to oxidation in GCMC simulations until achieving the Pt/O surface ratios listed in Table S1. Up to 1000 energy relaxation steps were performed between each GCMC step, with a convergence criterion of $0.5\,\mathrm{kcal\,mol^{-1}}$. To investigate energy barriers as a function of the Pt/O ratio, the 3 nm NP was oxidized to a Pt/O ratio of 1:1.5 at the surface. For all other simulations, oxidized structures with a Pt/O ratio of 1:1 at the NP surface were used for both the 2 and 3 nm NPs. To obtain the most stable NP structure, each oxidized NP underwent three annealing cycles. In the initial phase, the system was gradually heated from 300 K to the temperatures specified in Table S1 over 25 ps. The annealing temperature for each NP was chosen to allow structural rearrangements to achieve the most stable NP possible, while ensuring that no detachment of small clusters occurred, particularly for oxidized NPs. During the subsequent equilibration phase, the system was held at the designated temperature for 50 ps. The third phase involved gradual cooling of the systems back to 300 K over 50 ps, followed by a 5 ps equilibration period. This annealing process was repeated three times, with cooling the NP to 0 K in the final annealing cycle.

S12 Computational Cost

Most ReaxFF MD simulations in this work were performed using 16 CPU cores. The total number of atoms ranged up to approximately 25600, depending on NP size, oxidation state, graphite support, and presence of water. Simulation boxes varied from approximately $52 \times 51 \times 70\,\mathrm{\AA}^3$ (2 nm NPs) to $158 \times 137 \times 100\,\mathrm{\AA}^3$ (3 nm NPs). A fixed time step of 0.25 fs was used throughout all simulations. For structural preparation, each oxidized NP underwent three annealing cycles (390 ps) to obtain stable configurations (see Section S11). A second annealing step (180 ps) was used to anchor NPs on defective graphite supports in different orientations. Equilibration simulations were run for 200–250 ps, with energy convergence carefully monitored. Constrained MD simulations to study NP detachment typically lasted 100–250 ps, using a pulling velocity of 0.0001 Å/fs and continued until complete detachment was observed. These simulation timescales were sufficient to capture adsorption, restructuring, and detachment processes relevant to the studied systems.

References

[1] T. F. Miller, M. B. Hall, "Structural and Bonding Trends in PlatinumCarbon Clusters", *J. Am. Chem. Soc.* **1999**, *121*, 32, 7389.

[2] L. Gai, Y. K. Shin, M. Raju, A. C. T. van Duin, S. Raman, "Atomistic Adsorption of Oxygen and Hydrogen on Platinum Catalysts by Hybrid Grand Canonical Monte Carlo/Reactive Molecular Dynamics", *J. Phys. Chem. C* **2016**, *120*, 18, 9780.

[3] J. Bord, B. Kirchhoff, M. Baldofski, C. Jung, T. Jacob, "An Atomistic View of Platinum Cluster Growth on Pristine and Defective Graphene Supports", *Small* **2023**, *19*, 10, 2207484.

[4] D. Fantauzzi, J. Bandlow, L. Sabo, J. E. Mueller, A. C. T. van Duin, T. Jacob, "Development of a ReaxFF potential for Pt–O systems describing the energetics and dynamics of Pt-oxide formation", *Phys. Chem. Chem. Phys.* **2014**, *16,* 23118.

[5] B. Kirchhoff, L. Braunwarth, C. Jung, H. Jónsson, D. Fantauzzi, T. Jacob, "Simulations of the Oxidation and Degradation of Platinum Electrocatalysts", *Small* **2020**, *16*, 5, 1905159.

[6] D. Fantauzzi, S. Krick Calderón, J. E. Mueller, M. Grabau, C. Papp, H.-P. Steinrück, T. P. Senftle, A. C. T. van Duin, T. Jacob, "Growth of Stable Surface Oxides on Pt(111) at Near-Ambient Pressures", *Angew. Chem. Int. Ed.* **2017**, *56*, 10, 2594.

[7] D. Fantauzzi, J. E. Mueller, L. Sabo, A. C. T. van Duin, T. Jacob, "Surface Buckling and Subsurface Oxygen: Atomistic Insights into the Surface Oxidation of Pt(111)", *ChemPhysChem* **2015**, *16*, 13, 2797.

[8] Y. Wen, H. Fang, Z. Zhu, S. Sun, "Molecular dynamics investigation of shape effects on thermal characteristics of platinum nanoparticles", *Physics Letters A* **2009**, *373*, 2, 272.
